# Treatment Guidelines for PTSD: A Systematic Review

**DOI:** 10.3390/jcm10184175

**Published:** 2021-09-15

**Authors:** Alicia Martin, Mark Naunton, Sam Kosari, Gregory Peterson, Jackson Thomas, Julia K. Christenson

**Affiliations:** 1 Discipline of Pharmacy, Faculty of Health, University of Canberra, Canberra, ACT 2617, Australia; Mark.Naunton@canberra.edu.au (M.N.); Sam.Kosari@canberra.edu.au (S.K.); g.peterson@utas.edu.au (G.P.); Jackson.thomas@canberra.edu.au (J.T.); Julia.christenson@canberra.edu.au (J.K.C.); 2 School of Pharmacy and Pharmacology, University of Tasmania, Hobart, TAS 7001, Australia

**Keywords:** guidelines, PTSD, post-traumatic stress disorder, treatment, nightmares

## Abstract

Background: The aim of this review was to assess the quality of international treatment guidelines for post-traumatic stress disorder (PTSD), and identify differences between guideline recommendations, with a focus on the treatment of nightmares. Methods: Guidelines were identified through electronic searches of MEDLINE, CINAHL, PubMed, Embase and Science Direct, as well as web-based searches of international guideline repositories, websites of psychiatric organisations and targeted web-searches for guidelines from the three most populous English-speaking countries in each continent. Data in relation to recommendations were extracted and the AGREE II criteria were applied to assess for quality. Results: Fourteen guidelines, published between 2004–2020, were identified for inclusion in this review. Only five were less than 5 years old. Three guidelines scored highly across all AGREE II domains, while others varied between domains. Most guidelines consider both psychological and pharmacological therapies as first-line in PTSD. All but one guideline recommended cognitive behavioural therapy (CBT) as first-line psychological treatment, and selective serotonin reuptake inhibitors (SSRIs) as first-line pharmacological treatment. Most guidelines do not mention the targeted treatment of nightmares as a symptom of PTSD. Prazosin is discussed in several guidelines for the treatment of nightmares, but recommendations vary widely. Most PTSD guidelines were deemed to be of good quality; however, many could be considered out of date. Recommendations for core PTSD symptoms do not differ greatly between guidelines. However, despite the availability of targeted treatments for nightmares, most guidelines do not adequately address this. Conclusions: Guidelines need to be kept current to maintain clinical utility. Improvements are most needed in the AGREE II key domains of ‘applicability’, ‘rigour of development’ and ‘stakeholder involvement’. Due to the treatment-resistant nature of nightmares, guideline development groups should consider producing more detailed recommendations for their targeted treatment. More high-quality trials are also required to provide a solid foundation for making these clinical recommendations for the management of nightmares in PTSD.

## 1. Introduction

Post-traumatic stress disorder (PTSD) is a debilitating mental condition that can significantly impact the sufferer’s quality of life [1,2,3]. A study by Rapaport et al. found that 59% of patients suffering from PTSD had severely impaired overall quality of life based on the Quality of Life Enjoyment and Satisfaction Questionnaire [4]. Stein et al. found that 38.9% of patients with PTSD had missed at least one work day in the last month due to emotional problems, compared to only 5.4% of people who did not suffer from a mental health condition, and Kessler reported that a PTSD diagnosis increases the likelihood of being homeless by 150% [5,6]. PTSD is also commonly associated with comorbidities of depression and substance use disorders, and a significantly increased risk of suicide [7].

PTSD comprises four symptom clusters: ‘avoidance’, ‘numbing’, ‘hyper-arousal’ and the hallmark ‘re-experiencing’ or ‘intrusive symptoms’, which include unwanted thoughts, flashbacks and nightmares [8]. Nightmares are often resistant to general PTSD treatment and have been linked with a five-fold increase in suicidality [9]. Nightmares should therefore be considered one of the most important symptoms to treat, yet they are often overlooked as a secondary symptom of PTSD [10,11,12]. In addition, there appears to be few recommendations for the treatment of nightmares in guidelines, even though there are targeted treatments available, such as image rehearsal therapy (IRT) and pharmacotherapies including prazosin, terazosin and some atypical antipsychotics [13,14].

PTSD can be treated using psychological therapies, pharmacotherapy or a combination of the two. The recommended psychological therapies in Australia include trauma-focused cognitive behavioural therapy (CBT) and eye movement desensitisation reprocessing (EMDR) [15,16]. For pharmacotherapy in PTSD, the selective serotonin reuptake inhibitor (SSRI) antidepressants are recommended [15,16].

Evidence-based guidelines for the diagnosis and treatment of PTSD are valuable resources for psychiatrists and other health professionals to aid in the development of appropriate individual treatment plans for their patients, whilst also deterring the implementation of potentially ineffective or harmful treatments [17,18]. It can be difficult for healthcare professionals to keep up with and critically appraise the enormous volume of newly-published research in their field [19,20,21]. Evidence indicates that clinical psychiatric practice is often not in line with current guideline recommendations and hence may not reflect the best available evidence [22,23]. There are many barriers to implementing guidelines into practice. These include a perceived lack of time to review lengthy guidelines, an oversupply of guidelines (often with conflicting recommendations), a lack of usefulness in patients with significant comorbidities, a belief that current practice is best care based on past clinical experience, and an aversion to the perceived “rigidity” of guideline recommendations [19,21,22]. Every guideline is developed using different methodologies which can influence the quality of recommendations.

This review aimed to assess the quality of international treatment guidelines for PTSD, as well as identify differences between guideline recommendations, with a focus on the treatment of nightmares.

## 2. Materials and Methods

The Preferred Reporting Items for Systematic Reviews and Meta-Analysis (PRISMA) statement was followed to report the results of this review [24]. The review was registered with PROSPERO on the 21st of December 2017, registration number: CRD42017084122 [25].

### 2.1. Search Strategy

Relevant guidelines were identified via an electronic search of databases MEDLINE, CINAHL, PubMed, Embase and Science Direct, conducted in October 2017 using the search terms in Table 1. There were no date restrictions imposed; however, searches were restricted to the English language. A secondary search following the same methods was completed in September 2020 to identify any guidelines published after the initial search. Database searching was supplemented by web-based searches of guideline repositories (www.guidelines.gov, clinicalguidelines.gov.au and https://www.g-i-n.net/ (accessed date: 5 October 2017 and 21 September 2020), websites of international psychiatric organisations and targeted web-searches for guidelines from the three most populous English-speaking countries in each continent using combinations of the country names and search terms in Table 1. The latter was an additional step in an attempt to systematically capture any country-specific guidelines that were not listed in a guideline repository and may have otherwise been missed due to the search engine’s algorithm preferencing already identified guidelines. Reference lists of relevant guidelines were also searched manually for further relevant guidelines.

### 2.2. Selection Criteria

All treatment guidelines for PTSD or nightmares were considered for inclusion. Guidelines were excluded if they were specific to children or adolescents, were based entirely on other guidelines, did not make clear recommendations for treatments or were specific to complex PTSD. Complex PTSD is caused by exposure to an extreme or prolonged stressor from which escape is difficult or impossible (such as childhood abuse), presenting with a range of symptoms in addition to core PTSD symptoms, and for which there is a lack of available evidence for diagnosis and treatment [26].

Titles/abstracts of all identified records were reviewed and assessed for relevance by one researcher (AM). Full-text documents of relevant records were then obtained and reassessed against the inclusion criteria by the same researcher. Any concerns were rectified by consensus with two additional researchers (MN and SK).

### 2.3. Data Collection and Analysis

Data from relevant guidelines were independently extracted into a table by one researcher (AM). The following data were extracted: guideline title, author/institution, country, publication date, methodology for development of recommendations, first-line treatment recommendations and recommendations for the targeted treatment of PTSD-associated nightmares. No statistical analyses were completed.

### 2.4. Evaluation of Guideline Quality

The AGREE II instrument is a validated tool for the appraisal of health-related guidelines [27]. It consists of a checklist of 23 items grouped into six domains: scope and purpose; stakeholder involvement; rigour of development; clarity and presentation; applicability; and editorial independence. Each item is given a score from 1–7, with a score of 7 indicating that reporting of that item is exceptional and all criteria for the item have been met and a score of 1 indicating reporting of the item is absent or reporting criteria were not met. A quality score is then calculated for each of the 6 domains. This score is reported as a percentage of the maximum possible score for that domain and can then be used to interpret the strengths and limitations of the guideline, noting that there is no accepted cut-off score to define a ‘good’ guideline. The ‘Recommended for use?’ assessment (with options of Yes, No or Yes with modifications) is based on whether the guideline scored reasonably across all domains, the overall readability and usability of the guideline, and if the content is current. On completion of the AGREE II online tutorial, three reviewers (AM, JC & KM) independently assessed each guideline against the AGREE II criteria. They later met and agreed by consensus on the final averaged scores.

## 3. Results

**Figure 1:**
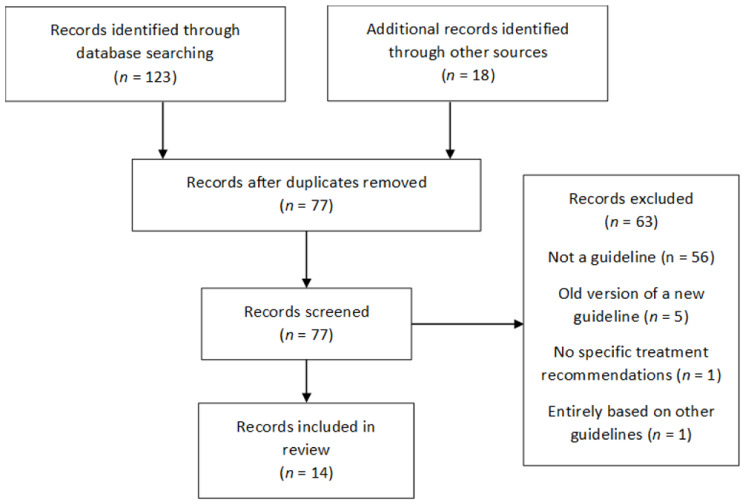
PRISMA Flow Diagram.

The search strategy identified a total of 133 records, of which, 14 guidelines matched the criteria for inclusion in this review. The secondary search in September 2020 identified updated versions of three previously identified guidelines. Reasons for exclusion of records are summarised in Figure 1. The general characteristics of each guideline are summarised in Table 2.

### 3.1. Guideline Characteristics

The fourteen guidelines identified in this review were published between 2004 and 2020, with only five less than 5 years old. There were four guidelines from the US, three from international organisations (the International Society for Traumatic Stress Studies, the World Federation of Societies of Biological Psychiatry and the World Health Organisation) two each from Australia, Canada and the UK, and one from South Africa. Their recommendations were based on evidence from conducting a systematic review, a consensus process, or a combination of the two.

### 3.2. Assessment of Guideline Quality

Domain scores and guideline abbreviations are presented in Table 3. All guidelines scored well in domain 1 ‘scope and purpose’. It had the second highest median score of 87% (range: 68–100%) and two guidelines (Phoenix & VA) scored 100% in this domain.

Scores in domain 2 ‘stakeholder involvement’ were more varied, with two guidelines scoring below 50% (AASM & WFSBP) yet four scoring above 90% (Phoenix, VA, APoA & NICE) (range 30–100%). Scores in domain 3 ‘rigour of development’ varied similarly to domain 2, with one guideline scoring below 50% (SASOP) and two above 90% (APoA & WHO) (Range: 44–92%). The highest scores were seen in domain 4 ‘clarity of presentation’, with a median score of 90% (range 71–100%). This is in contrast with domain 5 ‘applicability’, which saw high variability and a median score of just 49% (range 30–74%). Finally, domain 6 ‘editorial independence’ had the greatest variability between guidelines, with four scores of 90% or more (AASM, Phoenix, eTG and APoA) and four scores of 50% or less (CPA, WFSBP, ISTSS & SASOP) (range: 26–100%). Overall, the PTSD guidelines were of reasonably high quality; six are recommended for use, six are recommended with modifications, and two are not recommended for use after quality assessment (Table 3).

### 3.3. Recommendations in Guidelines

Five guidelines (36%) recommended that pharmacological interventions should be second-line to psychological interventions, while the others made no specific recommendation for one over the other. All fourteen guidelines (100%) recommended CBT (in various forms) as a first-line psychological treatment for PTSD. EMDR was also included as a first-line psychological option in 43%of the guidelines (Phoenix, BAP, WHO, eTG, NICE and ISTSS). All guidelines except one (93%), AASM, which was specific to treating nightmares, recommended an SSRI as first-line pharmacological treatment for PTSD if pharmacological treatment was indicated. Some recommended SSRIs broadly, while others specifically recommended only paroxetine, fluoxetine or sertraline. Most guidelines (71%) also included the serotonin-noradrenaline reuptake inhibitor (SNRI) venlafaxine as a first-line pharmacological treatment option. The WHO guideline also included tricyclic antidepressants (TCAs) as a first-line pharmacological recommendation.

Eight guidelines (57%) did not mention the targeted treatment of nightmares as a symptom of PTSD. Of the six guidelines (43%) that did mention targeted treatment, all mentioned prazosin as a potential option; although the strength of recommendations varied from no recommendation to first-line. Two guidelines (14%) also recommended IRT for targeted treatment of nightmares (AASM and APiA).

## 4. Discussion

To our knowledge, this review is the first study comparing international treatment guidelines for PTSD using a validated tool.

### 4.1. Quality of Guidelines

The AGREE II domains needing most attention were the critical ones of ‘applicability’, ‘rigour of development’ and ‘stakeholder involvement’. The Phoenix, APoA, WHO, NICE and VA guidelines received the highest scores across all domains (all >50%), while the ADAC and WFSBP guidelines received the lowest scores across all domains and were not recommended for use. Overall, guidelines scored well in the ‘scope and purpose’ and ‘clarity of presentation’ domains, consistently and clearly describing the guideline objectives and target populations. Scores in the stakeholder involvement domain varied. Low scores were generally due to a lack of information provided about the guideline development group members and/or a lack of consultation with patient representatives for their views and preferences.

No guidelines achieved a perfect score in the ‘rigour of development’ domain. While some guidelines scored highly, generally the external review process was missing entirely or lacked rigour. The SASOP guidelines scored particularly low in this domain, as the development process was not systematic. Each chapter was written by an expert or group of experts in the field, but there was no systematic evidence search described. However, this guideline was designed to be specific to a South African population, in which there is a lack of published evidence to review. Hence, a guideline based on experts’ clinical knowledge could be highly relevant, and still recommended for use despite low scores in some domains. All guidelines generally had lower scores in the applicability domain, while the editorial independence domain saw the greatest variance, with scores between 26–100%. Low scores in applicability were generally due to failure to identify barriers to implementation of the guidelines, as well as a lack of tools provided to aid implementation. In addition, very few guidelines performed economic evaluations to assess the potential resource implications of the application of their recommendations. While many guidelines scored highly in the ‘editorial independence’ domain, low scores were generally due to a lack of acknowledgement of the funding body or failure to state that no funding was received, highlighting a potential risk of bias in some guidelines.

The ‘rigour of development’ domain requires guideline developers to describe a procedure for updating the guideline. Seven guidelines (50%) described an intention for updates to occur. Some described a specific time interval (most commonly 5 years), while others stated updates were due only when new information becomes available. Interestingly, three of the remaining seven guidelines (50%) that did not describe an intention to update (BAP, ISTSS, VA) were updated versions of previous guidelines. While one could assume that one update means regular updates are planned, even updated guidelines should describe a procedure to be updated again, to remain valid. Of the seven guidelines (50%) that did describe an intention to be updated, two (eTG, WHO) are due for update based on the time interval they described. In addition, some guidelines that did not describe a time interval were likely to be due for update, although the ideal frequency of updates for clinical guidelines is unclear. A study by Shakelle et al. investigating how quickly clinical guidelines become outdated found that after 5.8 years, 50% of guidelines were no longer valid [40]. They concluded that all guidelines should generally be reassessed for validity at 3-year intervals [40]. However, a strict time interval recommendation is impractical in many cases, so the authors acknowledged that a 3-year interval will not always be suitable [40]. The evidence in certain fields is unlikely to change in 3 years, or even 10 years, while other rapidly evolving fields may require updates more frequently [40,41,42]. However, when making the recommendation to update a guideline “when new information becomes available” there must be a robust procedure in place to ensure new information is found and included in a timely manner to maintain the validity of the guideline [42].

### 4.2. Recommendations in Guidelines

SSRIs are universally accepted as first-line pharmacological treatment of PTSD in clinical guidelines. The evidence is strongest for paroxetine and fluoxetine, as well as the SNRI venlafaxine which is also commonly recommended as a first-line pharmacological treatment for PTSD [43]. A 2015 systematic review and meta-analysis of pharmacological treatment for PTSD found statistically significant improvements in PTSD symptom severity with paroxetine, fluoxetine and venlafaxine, when compared to placebo, with effect sizes similar to those seen for antidepressants in depression, although it should be noted these effect sizes are relatively small [43]. When all SSRIs were grouped together and compared to placebo, a small positive effect size was seen; however, based on current evidence it seems that greater benefit would be seen using either paroxetine or fluoxetine over other SSRIs [43].

TCAs were also recommended as a first-line pharmacological option in the WHO guideline, although it should be noted that pharmacological options were not recommended first-line unless CBT and EMDR are unavailable, have failed or there is severe comorbid depression. The evidence for TCAs is considered inferior to SSRIs and venlafaxine in most guidelines, and adverse effects are of greater concern. However, a recent systematic review of pharmacotherapy for combat-related PTSD found TCAs to have similar efficacy to SSRIs and, as such, more research in this area may be warranted [44].

Around one third of guidelines recommended psychotherapy over pharmacotherapy for first-line treatment of PTSD. This recommendation is supported by a recent meta-analysis comparing the two treatment approaches head to head, which found significantly greater effect sizes for trauma-focused psychotherapies compared to medications for treating PTSD [45]. However, this recommendation will not be appropriate for all patients; for example, cost of treatment may be a crucial factor for the patient, appropriate psychotherapy may not be readily available to patients in rural areas, patients may have comorbidities such as depression which could benefit from medications, or they may simply prefer medication over psychotherapy. In these situations, clinicians must use clinical judgement to determine the most appropriate course of action for the patient.

All guidelines included in this review described CBT as the first-line psychological treatment for PTSD. CBT is a broad term that can encompass several specific therapies, such as cognitive processing therapy (CPT), prolonged exposure therapy (PE) and image rehearsal therapy (IRT), which have a focus on cognitive, behavioural and emotional processing techniques [46]. Several guidelines use the term ‘trauma-focused CBT’, which tends to encompass CBT as well as trauma-focused psychotherapies, such as EMDR. However, some guidelines recommended EMDR as a separate therapy, which can be confusing when comparing recommendations [45]. A recent meta-analysis comparing CBT to EMDR found that EMDR was slightly superior to CBT for intrusive and arousal symptoms, but found no significant difference between groups for avoidance symptoms [47]. In addition, a systematic review of CBT for PTSD found that specific trauma-focused therapies were all superior to supportive, non-trauma-focused therapies [46]. Based on these findings, it is reasonable for guidelines to use the broad term “trauma-focused psychotherapies”, which includes CBT, CPT, PE and EMDR, as they all have comparable evidence for safety and efficacy, and the use of this term may reduce confusion while allowing for flexibility in treatment selection.

Eight (57%) of the guidelines (APoA, CPA, eTG, ISTSS, NICE, Phoneix and WFSBP, WHO) did not address the targeted treatment of nightmares, despite the fact that nightmares are often resistant to treatment and are associated with a significantly increased suicide risk [9,11,12]. Prazosin is recommended as first-line treatment for nightmares in PTSD in two guidelines (ADAC and AASM) while others recommended it as third-line therapy or included no specific recommendation but discussed potential for its use. In addition, the guidelines that did address targeted treatment often lacked significant depth and detail in both the evidence search and clarity of recommendations when compared to their recommendations for core PTSD symptoms. Three recently published meta-analyses investigating the efficacy of prazosin for PTSD nightmares found prazosin to be significantly more effective than placebo in reducing trauma-related nightmares [3,10,48]. These findings were based on improvements in the clinician-administered PTSD scale (CAPS) “distressing dreams item”, which is a measure of both the frequency and intensity of nightmares. Improvements were statistically significant and of large magnitude based on Cohen’s convention [3,10,48]. They did, however, highlight some important limitations, namely, small sample sizes and a lack of variability in both the research groups and participants, who were overwhelmingly male, combat veterans. Additionally, two more recent RCTs [49,50], not included in these meta-analyses, with relatively larger sample sizes, found no significant difference between prazosin and placebo in improving the CAPS distressing dreams item, or for any other primary outcome measures. While earlier positive trials for prazosin did not exclude participants with psychosocial instability, both negative studies excluded these patients. It is postulated that prazosin may only be effective in a sub-group of patients experiencing more severe adrenergic dysfunction, who may have been excluded from the negative studies [50,51]. While further research in this area is required, it remains important that guidelines consider the targeted treatment of nightmares in PTSD. Two guidelines also recommended IRT for targeted treatment of nightmares in PTSD. A recent systematic review and meta-analysis comparing IRT to prazosin found IRT to be equally effective as prazosin in treating nightmares in PTSD [14]. The most effective treatment, however, was IRT in combination with CBT for insomnia, which showed significant improvement in sleep quality when compared to prazosin or IRT alone (*p* = 0.03] [14]. It should be noted that there were significantly more trials investigating IRT than prazosin, and these included larger and more variable cohorts of participants, suggesting that the evidence for IRT is more robust than that for prazosin, yet IRT is recommended less frequently in treatment guidelines [14].

### 4.3. Strengths and Limitations

Strengths of this review include the use of the AGREE II criteria to assess the quality of each identified guideline, the development of a systematic method to search grey literature for guidelines, and a focus on the targeted treatment of nightmares. The AGREE II criteria is a validated assessment tool, which has been used in several similar systematic reviews of treatment guidelines in the area of mental health treatment [52,53,54]. It is also acknowledged that assessment using the AGREE II criteria can be relatively subjective, with no pre-defined cut-off scores.

Many systematic reviews of treatment guidelines do not clearly describe the method by which grey literature was searched to locate guidelines, or do not describe a systematic method that could be repeated by other researchers. Often, guidelines are not published in journals, and instead are posted on the webpages of the author organisation, or in guideline repositories, meaning that grey literature searches should form a major part of the search process [55]. Our grey literature search method was unique, transparent and systematic, and could be repeated by other researchers for future systematic reviews of treatment guidelines in any area of health care. This review also had a strong focus on the treatment of nightmares in PTSD, which are often resistant to general PTSD treatments [9]. Critical appraisal of the guidelines highlighted a lack of information about the targeted treatment of nightmares, despite the availability and importance of such treatments. A limitation of this review is the restriction to guidelines written in the English language, and although our method for searching the grey literature was highly systematic, it remains possible that eligible guidelines could have been missed.

## 5. Conclusions

There are a plethora of treatment guidelines being produced world-wide to aid health professionals in clinical decision making. However, it is important for health professionals to be able to identify which guidelines can be the most trusted. This review assessed the quality of international treatment guidelines for PTSD. We identified that many PTSD guidelines could be considered out of date require improvement in the AGREE II key domains of ‘applicability’, ‘rigour of development’ and ‘stakeholder involvement’. We have also highlighted a significant lack of information regarding the targeted treatment of nightmares, despite the availability of both psychological and pharmacological treatments. Due to the treatment-resistant nature of nightmares in PTSD and significantly increased risk of suicide, guideline development groups should consider producing more detailed recommendations for their treatment. In part, this is dependent on the availability of a solid evidence base if making specific treatment suggestions. Therefore, there is a need for more well-designed RCTs to establish the true clinical efficacy of targeted treatments for nightmares in PTSD.

## Figures and Tables

**Table 1:** Search Terms.

PTSD Terms	PTSD, Posttraumatic Stress Disorder, Post Traumatic Stress Disorder, Post-Traumatic Stress Disorder, Stress Disorders
Nightmares terms	Nightmares, sleep disturbance, sleep disruption
Guideline terms	Treatment guideline, practice guideline, evidence-based practice, best practice, consensus, clinical practice guideline, protocol
Three most populous English-speaking countries in each continent	Tanzania, Kenya, South Africa, India, Pakistan, Philippines, Australia, New Zealand, Papua New Guinea, England, Ireland, Scotland, The US, Canada, Jamaica

**Table 2:** Characteristics of included guidelines.

Guideline (Abbreviation)	Author/Institution	Country	Publication Date	First-Line Psychological Recommendation	First-Line Pharmacological Recommendation	Recommendation for Targeted Treatment of Nightmares (Y/N)	Recommendation for Use of Prazosin
Practice Guideline for the Treatment of Patients with Acute Stress Disorder and Posttraumatic Stress Disorder (APiA) [28]	American Psychiatric Association	United States	2004 + minor update 2009	CBT or EMDR	SSRIs	Y—IRT and prazosin suggested	“Low dose could be tried and increased if response is inadequate.”
Clinical Practice Guidelines for the Management of Anxiety Disorders (CPA) * [29]	Canadian Psychiatric Association	Canada	2006	CBT	Fluoxetine, paroxetine, sertraline, venlafaxine XR	N—But prazosin is included as a third-line adjunct option	Third-line as adjunct
World Federation of Societies of Biological Psychiatry Guidelines for the Pharmacological Treatment of Anxiety, Obsessive-Compulsive and Post-Traumatic Stress Disorders—First Revision (WFSBP) [30]	World Federation of Societies of Biological Psychiatry	International	2009	CBT	SSRIs (fluoxetine, sertraline, paroxetine), venlafaxine.	N—But prazosin is mentioned based on Category C evidence	Third-line
Best Practice Guide for the Treatment of Nightmare Disorder (AASM) [31]	American Academy of Sleep Medicine	United States	2010	CBT (IRT form specifically)	Prazosin	Y Prazosin & IRT = First-line, Level A	First-line—Level A
Guidelines for the Management of Conditions Specifically Related to Stress (WHO) [32]	World Health Organisation	International	2013	CBT or EMDR	*Second-line to psychological therapies* SSRIs, TCAs	N	No recommendation
eTG complete—Posttraumatic Mental Health Disorders (eTG) [15]	Therapeutic Guidelines Ltd.	Australia	2013	CBT or EMDR	*Second-line to psychological therapies* SSRIs	N	No recommendation
The South African Society of Psychiatrists (SASOP) Treatment Guidelines for Psychiatric Disorders (SASOP) [33]	The South African Society of Psychiatrists	South Africa	2013	CBT	SSRIs and SNRIs	Y	“Prazosin has shown promise in treating nightmares”
Canadian Clinical Practice Guidelines for the Management of Anxiety, Posttraumatic Stress and Obsessive-compulsive Disorders (ADAC) [34]	Anxiety Disorders Association of Canada	Canada	2014	Trauma-focused CBT or EMDR	Paroxetine, venlafaxine	Y	Level 1 for nightmares
Evidence-based Pharmacological Treatment of Anxiety Disorders, Post-traumatic Stress Disorder and Obsessive-compulsive Disorder: A Revision of the 2005 Guidelines from the British Association for Psychopharmacology (BAP) [35]	British Association for Psychopharmacology	United Kingdom	2014	Trauma-focused CBT or EMDR	Paroxetine, sertraline, venlafaxine	Y	As adjunct if initial treatment fails
VA/DOD Clinical Practice Guideline for the Management of Posttraumatic Stress Disorder and Acute Stress Disorder (VA) [36]	Department of Veterans Affairs and Department of Defense	United States	2017	Manualised trauma-focused CBT (including EMDR)	*Second-line to psychological therapies* SSRIs (fluoxetine, sertraline, paroxetine), venlafaxine.	Y	Insufficient evidence for or against its use
Clinical Practice Guidelines for the Treatment of PTSD (APoA) [37]	American Psychological Association	United States	2017	CPT, CT, Trauma-focused CBT, and PE	SSRIs (fluoxetine, sertraline, paroxetine), venlafaxine.	N	No recommendation
Post Traumatic Stress Disorder NICE Guidance (NICE) [38]	National Institute for Clinical Excellence	United Kingdom	2018	Trauma-focused CBT	Venlafaxine or SSRIs *only if person has preference for drug treatment*	N	No recommendation
Effective Treatments for PTSD: Third Edition (ISTSS) [39]	International Society for Traumatic Stress Studies	International	2020	CPT, CT, Trauma-focused CBT, EMDR and PE	SSRIs (fluoxetine, sertraline, paroxetine), venlafaxine.	N	No recommendation
Australian Guidelines for the Prevention and Treatment of Acute Stress Disorder, Posttraumatic Stress Disorder, and Complex Posttraumatic Stress Disorder (Phoenix) [16]	Phoenix Australia—Centre for Posttraumatic Mental Health	Australia	2020	CPT, CT, Trauma-focused CBT, EMDR and PE	*Second-line to psychological therapies* SSRIs (sertraline, fluoxetine, paroxetine), venlafaxine	N	No recommendation

CBT = cognitive behaviour therapy, CPT = cognitive processing therapy, CT = cognitive therapy, EMDR = eye movement desensitisation and reprocessing, IRT = image rehearsal therapy, PE = prolonged exposure, SNRI = serotonin-norepinephrine reuptake inhibitor, SSRI = selective serotonin reuptake inhibitor, TCA = tricyclic antidepressant. * This guideline has not been reviewed since its publication in 2006. It is the policy of the Canadian Psychiatric Association to review clinical practice guidelines every 5 years, and any document that does not explicitly state that it has been reviewed should be considered as a historical document only. It is included in this review for interest, but we acknowledge that it is not considered current guidance.

**Table 3:** AGREE II assessment scores * for each guideline.

Guideline (Abbreviation)	Domain 1 *Scope and Purpose*	Domain 2 *Stakeholder Involvement*	Domain 3 *Rigour of development*	Domain 4 *Clarity of presentation*	Domain 5 *Applicability*	Domain 6 *Editorial independence*	Recommended for Use?
Practice Guideline for the Treatment of Patients with Acute Stress Disorder and Posttraumatic Stress Disorder (APiA) [28]	68%	52%	66%	71%	42%	79%	Y/M
Clinical Practice Guidelines for the Management of Anxiety Disorders (CPA) [29]	81%	56%	57%	86%	42%	43%	Y/M
World Federation of Societies of Biological Psychiatry Guidelines for the Pharmacological Treatment of Anxiety, Obsessive-Compulsive and Post-Traumatic Stress Disorders—First Revision (WFSBP) [30]	76%	44%	60%	71%	37%	50%	N
Best Practice Guide for the Treatment of Nightmare Disorder (AASM) [31]	89%	30%	85%	90%	30%	93%	Y/M
Guidelines for the Management of Conditions Specifically Related to Stress (WHO) [32]	98%	73%	92%	100%	67%	86%	Y
eTG complete—Posttraumatic Mental Health Disorders (eTG) [15]	68%	68%	55%	83%	37%	93%	Y/M
The South African Society of Psychiatrists (SASOP) Treatment Guidelines for Psychiatric Disorders (SASOP) [33]	79%	67%	44%	89%	49%	43%	Y
Canadian Clinical Practice Guidelines for the Management of Anxiety, Posttraumatic Stress and Obsessive-compulsive Disorders (ADAC) [34]	71%	57%	54%	71%	31%	71%	N
Evidence-based Pharmacological Treatment of Anxiety Disorders, Post-traumatic Stress Disorder and Obsessive-compulsive Disorder: A Revision of the 2005 Guidelines from the British Association for Psychopharmacology (BAP) [35]	87%	76%	60%	95%	48%	76%	Y/M
VA/DOD Clinical Practice Guideline for the Management of Posttraumatic Stress Disorder and Acute Stress Disorder (VA) [36]	100%	100%	84%	100%	67%	52%	Y
Clinical Practice Guidelines for the Treatment of PTSD (APoA) [37]	98%	100%	92%	97%	60%	100%	Y
Post Traumatic Stress Disorder NICE Guidance (NICE) [38]	95%	100%	86%	98%	61%	64%	Y
Effective Treatments for PTSD: Third Edition (ISTSS) ** [39]	86%	70%	64%	78%	57%	26%	Y/M
Australian Guidelines for the Treatment of Acute Stress Disorder & Posttraumatic Stress Disorder (Phoenix) [16]	100%	93%	89%	92%	74%	95%	Y
*Domain median (range)*	*87%* *(68–100%)*	*69%* *(30–100%)*	*65%* *(44–92%)*	*90%* *(71–100%)*	*49%* *(30–74%)*	*74%* *(26–100%)*	

Y/M = Yes with modifications. * Domain 1. Scope and Purpose is concerned with the overall aim of the guideline, the specific health questions, and the target population (items 1–3). Domain 2. Stakeholder Involvement focuses on the extent to which the guideline was developed by appropriate stakeholders and represents the views of its intended users (items 4–6). Domain 3. Rigour of Development relates to the process used to gather and synthesise the evidence, the methods to formulate the recommendations, and to update them (items 7–14). Domain 4. Clarity of Presentation deals with the language, structure, and format of the guideline (items 15–17). Domain 5. Applicability pertains to the likely barriers and facilitators to implementation, strategies to improve uptake, and resource implications of applying the guideline (items 18–21). Domain 6. Editorial Independence is concerned with the formulation of recommendations not being unduly biased with competing interests (items 22–23). ** The ISTSS guidelines are published as a book for purchase. The Methodology and Recommendations document is available publicly on the internet, but the Evidence Summary documents, and reference lists are available for members only. These documents were requested but not supplied, so it is likely the ISTSS guideline would have scored higher in certain categories if these documents were available.

## Data Availability

The data presented in this study are available within the article.
